# Health-related quality of life in survivors of stage I-II breast cancer: randomized trial of post-operative conventional radiotherapy and hypofractionated tomotherapy

**DOI:** 10.1186/1471-2407-12-495

**Published:** 2012-10-25

**Authors:** Harijati Versmessen, Vincent Vinh-Hung, Hilde Van Parijs, Geertje Miedema, Mia Voordeckers, Nele Adriaenssens, Guy Storme, Mark De Ridder

**Affiliations:** 1 Department of Radiation Oncology, UZ Brussel, Vrije Universiteit Brussel, Laarbeeklaan 101, 1090, Jette, Brussels, Belgium; 2 Radiation oncology, Geneva University Hospital, Geneva, Switzerland; 3 Department of Physical Therapy, UZ Brussel, Breast Clinic, Brussels, Belgium; 4 Department of Physical Therapy, Vrije Universiteit Brussel, Brussels, Belgium

**Keywords:** Health-related quality of life, Breast cancer, Hypofractionated radiotherapy, Adjuvant treatment, Randomized trial

## Abstract

**Background:**

Health-related quality of life (HRQOL) assessment is a key component of clinical oncology trials. However, few breast cancer trials comparing adjuvant conventional radiotherapy (CR) and hypofractionated tomotherapy (TT) have investigated HRQOL. We compared HRQOL in stage I-II breast cancer patients who were randomized to receive either CR or TT. Tomotherapy uses an integrated computed tomography scanner to improve treatment accuracy, aiming to reduce the adverse effects of radiotherapy.

**Methods:**

A total of 121 stage I–II breast cancer patients who had undergone breast conserving surgery (BCS) or mastectomy (MA) were randomly assigned to receive either CR or TT. CR patients received 25 × 2 Gy over 5 weeks, and BCS patients also received a sequential boost of 8 × 2 Gy over 2 weeks. TT patients received 15 × 2.8 Gy over 3 weeks, and BCS patients also received a simultaneous integrated boost of 15 × 0.6 Gy over 3 weeks. Patients completed the EORTC QLQ-C30 and BR23 questionnaires. The mean score (± standard error) was calculated at baseline, the end of radiotherapy, and at 3 months and 1, 2, and 3 years post-radiotherapy. Data were analyzed by the 'intention-to-treat' principle.

**Results:**

On the last day of radiotherapy, patients in both treatment arms had decreased global health status and functioning scores; increased fatigue (clinically meaningful in both treatment arms), nausea and vomiting, and constipation; decreased arm symptoms; clinically meaningful increased breast symptoms in CR patients and systemic side effects in TT patients; and slightly decreased body image and future perspective.

At 3 months post-radiotherapy, TT patients had a clinically significant increase in role- and social-functioning scores and a clinically significant decrease in fatigue. The post-radiotherapy physical-, cognitive- and emotional-functioning scores improved faster in TT patients than CR patients. TT patients also had a better long-term recovery from fatigue than CR patients. ANOVA with the Bonferroni correction did not show any significant differences between groups in HRQOL scores.

**Conclusions:**

TT patients had a better improvement in global health status and role- and cognitive-functioning, and a faster recovery from fatigue, than CR patients. These results suggest that a shorter fractionation schedule may reduce the adverse effects of treatment.

## Background

Breast cancer is the most commonly occurring cancer in women [1]. Worldwide, breast cancer accounted for 23% of new cancer cases and 14% of total cancer deaths in 2008 [2]. Radiotherapy is standard treatment in all patients who undergo breast conserving surgery (BCS), and also plays a major role in the treatment of patients who undergo mastectomy (MA) [3]. Adjuvant radiotherapy has been shown to improve local control and overall survival, with a 70% reduction in the risk of recurrence [4,5] and a 9–12% reduction in the risk of death [6-9]. These improved survival rates are based on trials of conventional protocols in which 1.8–2.5 Gy/fraction was delivered over 5–7 weeks [6,8,10-12]. There has been concern that delivery of > 2 Gy/fraction might increase late toxicity and impair cosmesis in BCS patients [13]. It is known that the late effects are strongly dependent on dose per fraction, with higher doses per fraction resulting in a greater susceptibility of healthy tissues to the adverse effects of radiotherapy. The Early Breast Cancer Trialists' Collaborative Group reported that radiotherapy using conventional fractionation reduced the annual mortality rate of breast cancer patients by 13%, but increased the annual mortality rate due to other causes by 21%, and that this increase was due primarily to cardiovascular effects [14]. A hypofractionated schedule has the potential to result in even more severe adverse effects.

Many researchers are investigating hypofractionated radiotherapy for breast cancer, aiming to determine the optimal schedule for cosmesis, late toxicity, and locoregional control. Most of the randomized trials that compare conventional radiotherapy (CR) with hypofractionated radiotherapy have reported on effectiveness (locoregional control) and safety (acute and late toxicity) [15-24]. However, only a few studies have investigated cosmesis [15,19,20], and only one study to date has investigated quality of life (QOL) [19].

Health-related QOL (HRQOL) assessment is now regarded as a key component of clinical oncology trials [25]. Radiotherapy for breast cancer tends to be stressful and may increase fatigue, skin irritation, and breast pain during the first year [26]. Attendance at daily radiotherapy treatments for up to 6 weeks may also have an impact on the patient's QOL. It is hoped that use of the hypofractionated schedule can reduce this burden by shortening the overall treatment time.

Sprangers [27] considered that HRQOL can be measured reliably and validly, and that measurement of HRQOL helps clinicians to gain insight into patients’ perspectives of their disease and treatment. However, patients may change their perspectives during the course of their disease experience, referred to as a ‘response shift.’ This may result in patients reporting a stable QOL over time in standardized questionnaires, while concurrently exhibiting deteriorating clinical health [28,29].

Tomotherapy is a new radiotherapy system that uses an integrated computed tomography scanner to improve the accuracy of radiotherapy treatment. The radiation is delivered helicoidally, allowing highly conformal shaping of dose distribution while minimizing radiation exposure to healthy tissues. However, the magnitude of the clinical advantage of using this system in breast cancer treatment is currently unknown. We therefore designed a randomized phase III trial to compare CR with hypofractionated tomotherapy (TT), using the Tomotherapy® system (NCT00459628). The primary endpoint of the trial was pulmonary or cardiac toxicity, and the secondary endpoint was locoregional recurrence. Completion of HRQOL questionnaires (EORTC QLQ C-30 & BR-23) was included in the trial design. The purpose of this paper is to compare the HRQOL questionnaire results between the two treatment arms.

## Methods

Breast cancer patients who underwent surgery at the University Hospital of Brussels from June 2007 to July 2011 were screened according to the eligibility criteria in the protocol of the TomoBreast study (ClinicalTrials.gov registration NCT00459628):

1. Women aged 18 years or older.

2. Histologically proven invasive unilateral breast carcinoma, stage I or II (T1-3N0 or T1-2N1 M0, American Joint Committee on Cancer (AJCC)/TNM 6th edition).

3. BCS or MA with clear margins and pathological nodal status assessed by axillary lymph node dissection or sentinel node biopsy.

4. At least one pre-operative medical imaging scan available (computed tomography, magnetic resonance imaging, or positron emission tomography).

5. Informed consent obtained.

Patients who did not meet the inclusion criteria, or with the following criteria, were excluded:

1. Prior breast or thoracic radiotherapy.

2. Pregnancy or lactation.

3. Fertile without effective contraception.

4. Psychiatric or addictive disorder.

**Figure 1:**
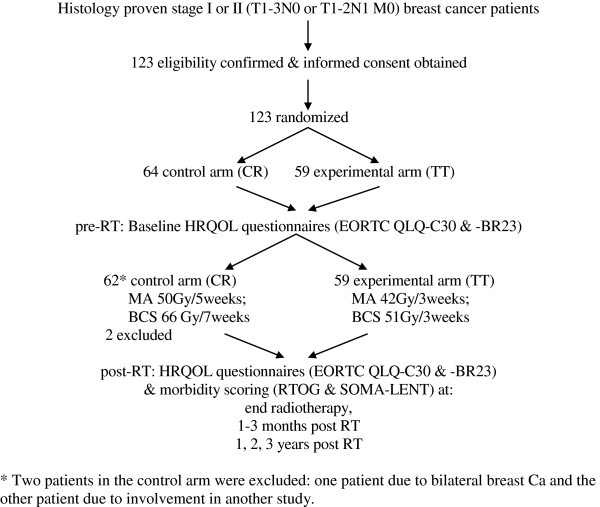
Participant flow.

A total of 123 eligible patients gave written informed consent and were included in the study. These patients were randomized to the CR (control) or TT (experimental) arms using Efron's biased coin design [30]. Patients were stratified by nodal status (N0 vs. N1), type of surgery (MA vs. BCS), and chemotherapy sequence (none vs. sequential vs. concomitant chemotherapy). Two patients who were randomized to the control arm were later excluded from the study. One of these patients had bilateral breast cancer, which was not in accordance with the eligibility criteria, and the other patient could not participate because she was enrolled in a different study. The participant flow chart is presented in Figure 1. In November 2011, the 121 eligible patients had all been followed up for at least 3 months after the completion of radiotherapy. 

CR patients received a dose of 50 Gy delivered in 25 fractions over 5 weeks to the chest wall using tangential photon fields, and in patients with pN1 status, to the supraclavicular, infraclavicular, and axillary nodes using an anterior field matched to the tangential fields. BCS patients received a sequential boost of 16 Gy delivered in 8 fractions over 2 weeks to the initial tumor bed using a direct electron field (cumulative dose 66 Gy over 6.5 or 7 weeks depending on maintenance procedures). TT patients received a dose of 42 Gy delivered in 15 fractions over 3 weeks to the chest wall of MA patients or to the whole breast of BCS patients, and to the supraclavicular, infraclavicular, and axillary nodes in patients with pN1 status, using the image-guided Tomotherapy® system. BCS patients received a simultaneous integrated boost of 9 Gy delivered in 15 fractions over the 3 weeks (cumulative dose 51 Gy over 3 weeks).

Concurrent or sequential adjuvant systemic treatments were allowed. According to the protocol, radiotherapy should start within 6 weeks after breast surgery, or in cases of sequential chemotherapy, within 6 weeks after the completion of chemotherapy (Table 1). In reality, CR started an average of 39 days after surgery and TT started an average of 50 days after surgery in patients who did not receive chemotherapy. CR started an average of 43 days after surgery and TT started an average of 49 days after surgery in patients with concurrent chemotherapy. One patient who received neo-adjuvant chemotherapy received radiotherapy 36 days after surgery. Patients with sequential chemotherapy started CR an average of 23 days, or TT an average of 25 days, after the completion of chemotherapy.

**Table 1:** Mean nr of days to start RT after last breast surgery or last chemotherapy

		**CR**	**TT**
**no adj CT**	**nr of pts**	37	29
	**after last breast surgery**	39	50
**neo**-**adj CT**	**nr of pts**	-	1
	**after last breast surgery**	-	36
**concurrent CT**	**nr of pts**	19	23
	**after last breast surgery**	43	49
**sequential CT**	**nr of pts**	6	6
	**after last breast surgery**	164	154
	**after last CT**	23	25

The European Organisation for Research and Treatment of Cancer (EORTC) general cancer quality of life score (QLQ-C30) questionnaire and its breast cancer module (QLQ-BR23) were used to measure HRQOL in this study. These questionnaires were specifically designed for cancer patients, have undergone extensive testing, and have been confirmed as reliable and valid when measuring QOL outcomes [31,32]. The EORTC QLQ-C30 questionnaire consists of 30 questions which assess functioning (physical, role, cognitive, emotional, social) and symptoms (fatigue, nausea and vomiting, pain, dyspnea, insomnia, appetite loss, constipation, diarrhea, financial difficulty), and a global health status score that assesses overall QOL. The EORTC QLQ-BR23 questionnaire consists of 23 questions assessing functioning (body image, sexual functioning, sexual enjoyment, future perspective) and symptoms (systemic side effects, upset by hair loss, breast symptoms, arm symptoms). Both questionnaires use a four-point response scale (not at all, a little, quite a bit, and very much) to assess each functional or symptom item, and a seven-point response scale is used to assess global health status (from very poor to excellent). Raw scores were linearly transformed into a score of 0–100 for processing according to the EORTC manual [33]. Higher scores in the functioning and global health status scales represented better functioning and QOL, whereas higher scores in the symptom scales indicated greater problems.

Patients completed the HRQOL questionnaires (EORTC QLQ-C30 and BR-23) during hospital visits at baseline (prior to radiotherapy), on the last day of radiotherapy, at 1–3 months after the completion of radiotherapy, and then yearly for 3 years. Clinical evaluations were performed at the same time points, and any recurrence of cancer was documented. The Radiation Therapy Oncology Group (RTOG)/EORTC morbidity scoring schema [34] was used to assess acute morbidity, and the RTOG/EORTC and the Subjective Objective Management Analytic/Late Effects on Normal Tissues (SOMA/LENT) toxicity scales [35] were used to assess late morbidity.

Patients usually completed the HRQOL questionnaires during their hospitals visits, but if they did not have time, they were asked to return them by mail. This achieved a 100% return rate at all time points except on the last day of radiotherapy (96% compliance), when five patients (two CR patients and three TT patients) declined to complete the questionnaires for various reasons (inconvenient, too busy, too tired, etc.). Six patients (two CR patients and four TT patients) withdrew from the study for various reasons (the patient did not want to undergo all the tests, the hospital was too far from the home, the family was not available to accompany the patient for hospital visits). These patients therefore did not complete the HRQOL questionnaires after their withdrawal from the study: one TT patient withdrew at the end of radiotherapy, one CR patient withdrew at 3 months after radiotherapy, one CT patient withdrew at 1 year, two TT patients withdrew at 2 years, and one TT patient withdrew at 3 years.

The mean (± standard error) of each score was calculated at each time point: baseline, last day of radiotherapy, 3 months, and 1, 2, and 3 years after the completion of radiotherapy. Consistent with previous studies, only differences of greater than ten points on the transformed questionnaire scale were considered clinically meaningful [36-38].

Data were analyzed by the intention-to-treat (ITT) principle. For each patient, the baseline HRQOL score was subtracted from the score at each subsequent time point. The average change at each time point was compared between treatment arms using the two-sample *t*-test (Additional file 1: adjusted QLQ mean scores.xls). A positive change indicated improvement of functioning or worsening of symptoms, and a negative change indicated worsening of functioning or improvement of symptoms. Proportions were compared using Fisher's exact test and mean scores were compared using the t-test (two-sided), with the level of significance set at p < 0.05. Mean scores were also compared using the Bonferroni correction and repeated measures ANOVA. Statistical analyses were conducted using JMP version 8.0.1 (SAS Institute Inc., Cary, NC, USA).

### Patient characteristics

Efron's biased coin design was used to randomize patients to treatment arms [30]. Patients in each treatment arm (CR and TT) were stratified by nodal status, type of surgery, and chemotherapy sequence. The baseline patient and tumor characteristics, adjuvant radio-chemotherapy schedules, and hormonal treatments are presented in Table 2. 

**Table 2:** Baseline characteristics

**N (%)**	**CR (n=62)**	**TT (n=59)**
**Age**		
Mean age at randomization (SD)	58 (11)	55 (11)
>/=65 years old	21 (34)	13 (22)
> 65 years old	41 (66)	46 (78)
**Surgery**		
Mastectomy	19 (31)	26 (31)
Segmentectomy	43 (69)	33 (69)
Axillary nodes	10 (16)	16 (27)
Sentinel nodes	43 (69)	30 (51)
Sentinel & axillary nodes	9 (15)	13 (22)
**Tumor grade** &**nodal status**		
T1	38 (61)	39 (66)
T2	24 (39)	20 (34)
N0	46 (74)	38 (64)
N1, LNR 0.01-0.20	11 (69)	18 (86)
N1, LNR 0.21-0.65	5 (31)	3 (14)
N1, LNR >0.65	0	0
**Side**		
Right	30 (48)	24 (41)
Left	32 (52)	35 (59)
**Mean size of largest tumor** (**mm**) (SD)		
T1 (<=20 mm)	12,5 (4,8)	13,4 (4,9)
T2 (21–50 mm)	25,4 (6,9)	27,5 (5,9)
**Quadrant**		
Central	6 (10)	9 (15)
Supero-interne	12 (19)	10 (17)
Infero-interne	9 (15)	1 (2)
Supero-externe	21 (33)	32 (54)
Infero-externe	6 (10)	4 (7)
Overlapping	5 (8)	2 (3)
>/= 2 locations	3 (5)	1 (2)
**Histology grade**		
1	17 (27)	16 (27)
2	25 (40)	29 (49)
3	16 (26)	12 (20)
Unknown	4 (7)	2 (4)
**Estrogen positive**	54 (87)	48 (81)
**Progesterone positive**	45 (73)	46 (78)
**Her2 FISH positive**	3 (5)	10 (17)
**Adjuvant radio**-**chemotherapy** (**RT**-**CT**) **schedule**		
No CT	37 (60)	29 (49)
RT concurrent with CT	19 (30)	23 (39)
RT after CT (sequential)* (one patient neo-adj CT)	6 (10)	7 (12)*
**Chemotherapy type**		
Anthracycline without taxane	4 (16)	5 (17)
Anthracycline with taxane	16 (64)	19 (63)
CMF	2 (8)	2 (7)
Anthracycline with taxotere	1 (4)	3 (10)
TCH	2 (8)	1 (3)
**Hormonal therapy** (**HT**)		
No HT	9 (14)	11 (19)
Tamoxifen	26 (42)	16 (28)
Femara	24 (39)	22 (38)
Zoladex	0	2 (3)
Tamoxifen + zoladex	3 (5)	6 (10)
Femara + zoladex	0	1 (2)
**Herceptin** (**Trastuzumab**)	3 (5)	10 (17)

### Baseline quality of life scores

The mean baseline scores of the EORTC QLQ-C30 and BR-23 questionnaires in each treatment arm are shown in Table 3. There were no significant differences in any of the scores between treatment arms at baseline. Only eight CR patients and 13 TT patients had hair loss at baseline. Of these, two CR patients and five TT patients who had received adjuvant chemotherapy before the start of radiotherapy described the hair loss as "very much" at baseline, and the other patients with hair loss due to other reasons described it as "quite a bit" at baseline. Some patients did not answer the questions about sexual functioning and enjoyment for personal reasons (such as religion or being widowed).

**Table 3:** **Baseline mean scores** (**SD**) **by treatment arm**

**EORTC**-**QLQ C30**	**CR**	**TT**
	**(n=62)**	**(n=59)**
physical functioning	84,1 (18,7)	83,2 (16,0)
role functioning	70,2 (27,4)	66,4 (29,3)
cognitive functioning	86,0 (20,5)	82,8 (22,3)
emotional functioning	78,8 (18,1)	74,4 (20,0)
social functioning	80,6 (22,6)	82,2 (19,8)
fatigue	29,7 (20,7)	35,0 (24,9)
nausea & vomiting	7,5 (19,0)	5,1 (15,2)
pain	24,7 (24,7)	24,5 (24,4)
global health status	69,0 (21,7)	67,2 (17,5)
dyspnea	11,3 (22,5)	15,3 (26,5)
insomnia	26,9 (28,2)	35,0 (29,3)
loss of appetite	12,9 (27,2)	10,2 (18,8)
obstipation	12,4 (25,8)	11,3 (18,2)
diarrhea	6,5 (16,9)	4,0 (12,5)
financial difficulty	9,7 (24,4)	13,0 (24,8)
**EORTC**-**QLQ BR23**	**CR**	**TT**
	**(n=62)**	**(n=59)**
systemic treatment side effects	13,9 (14,2)	15,4 (16,0)
body image	73,7 (28,6)	73,0 (30,9)
future perspective	52,7 (29,9)	54,2 (29,0)
arm symptoms	23,8 (22,6)	24,9 (21,6)
breast symptoms	21,9 (18,6)	19,9 (16,6)
	**CR** (**n**=**8**)	**TT** (**n**=**13**)
upset by hair loss	33,3 (35,6)	35,9 (39,6)
	**CR** (**n**=**56**)	**TT** (**n**=**54**)
sexual functioning	22,3 (23,2	25,0 (23,3)
	**CR** (**n**=**28**)	**TT** (**n**=**33**)
sexual enjoyment	56,0 (28,8)	55,6 (28,5)

## Results

**Figure 2:**
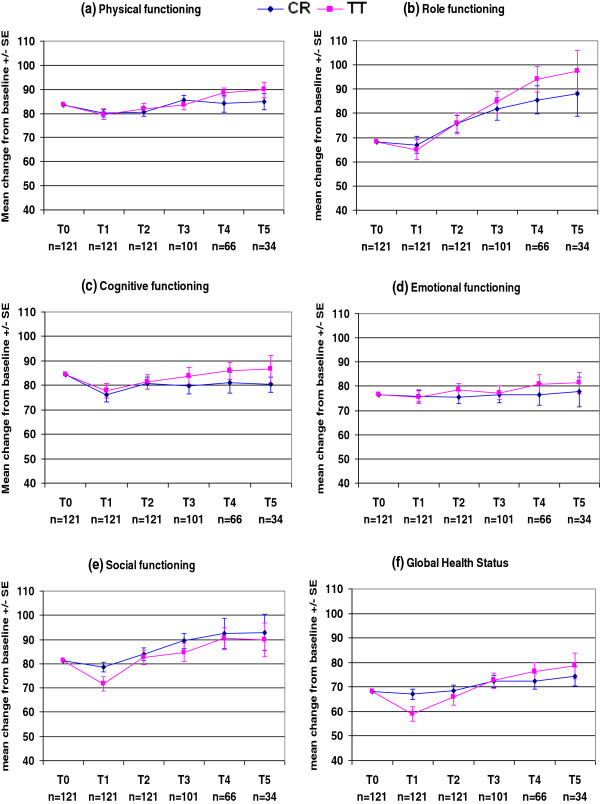
**EORTC QLQ**-**C30.**

**Figure 3:**
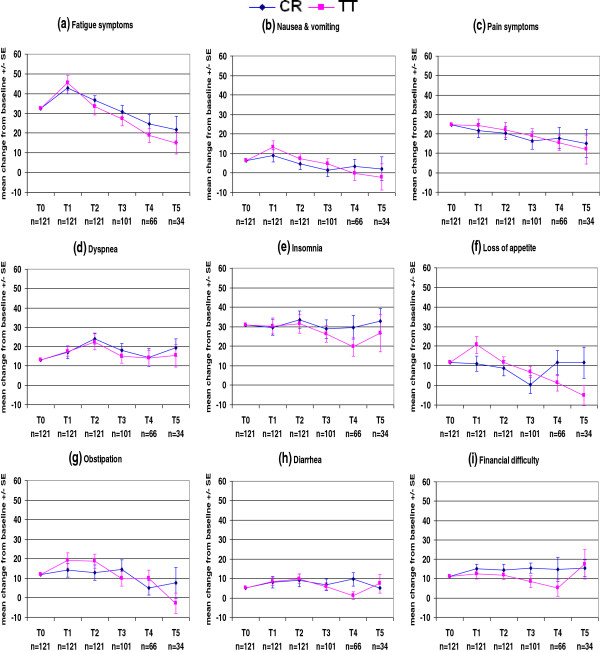
EORTC QLQ-C30.

**Figure 4:**
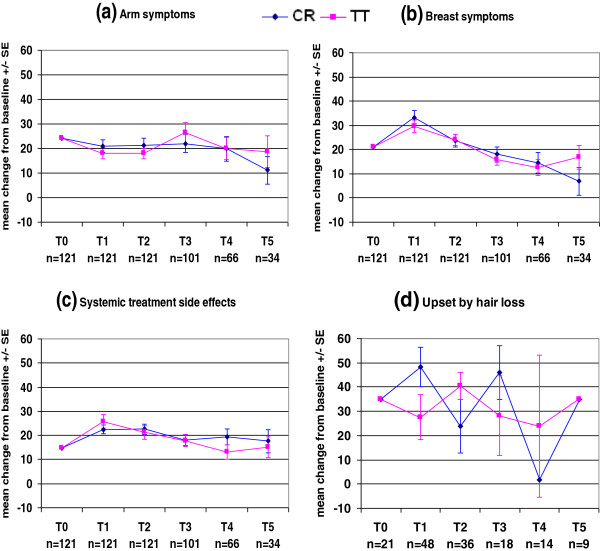
EORTC QLQ-BR23.

**Figure 5:**
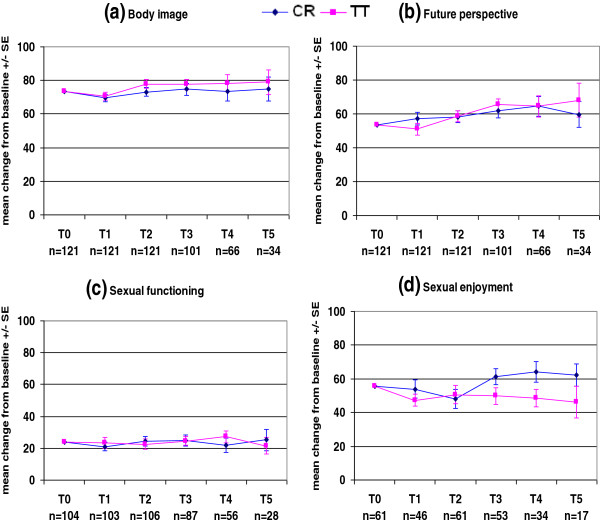
EORTC QLQ-BR23.

The QLQ-C30 and QLQ-BR23 mean scores at each time point in each treatment arm are presented in Figures 2, 3, 4 and 5 and Tables 4 and 5.

**Table 4:** **EORTC QLQ**-**C30 mean scores** (**SE**) **at each time point**

	**CR &TT at T0**	**CR at T1**	**TT at T1**	**CR at T2**	**TT at T2**	**CR at T3**	**TT at T3**	**CR at T4**	**TT at T4**	**CR at T5**	**TT at T5**
	**n**=**121**	**n**=**62**	**n**=**59**	**n**=**62**	**n**=**59**	**n**=**50**	**n**=**51**	**n**=**30**	**n**=**36**	**n**=**16**	**n**=**18**
**physical functioning**	83,64	80,08 (1,64)	79,39 (2,03)	80,69 (1,70)	82,03 (2,18)	85,44 (1,96)	83,64 (1,97)	84,08 (3,50)	88,69 (1,88)	84,89 (3,29)	89,89 (3,19)
**role functioning**	68,32	66,93 (3,51)	64,99 (4,22)	75,70 (3,45)	75,79 ^a^ (4,26)	81,86 (4,62)	84,65 (4,45)	85,54 (5,73)	94,08 (5,38)	88,11 (9,41)	97,49 (8,67)
**cognitive functioning**	84,44	76,10 (2,82)	77,77 (3,02)	80,88 (2,50)	81,27 (2,94)	79,92 (3,60)	83,77 (3,40)	81,10 (4,26)	85,95 (3,51)	80,27 (3,23)	86,52 (5,67)
**emotional functioning**	76,65	75,96 (2,50)	75,44 (2,60)	75,56 (2,60)	78,52 (2,74)	76,65 (3,46)	77,32 (2,80)	76,65 (4,36)	80,69 (4,14)	77,69 (6,17)	81,34 (4,50)
**social functioning**	81,40	78,63 (2,10)	71,71 (3,08)	83,86 (2,64)	82,55 ^a^ (2,89)	89,39 (3,25)	84,74 (3,69)	92,52 (6,16)	90,50 (4,48)	92,86 (7,41)	89,74 (6,97)
**global health status**	68,11	67,00 (2,22)	59,02 (2,90)	68,52 (2,24)	65,81 (3,09)	72,28 (2,48)	72,61 (3,14)	72,28 (3,22)	76,19 (3,78)	74,36 (4,06)	78,53 (5,30)
**fatigue**	32,32	42,88 ^a^ (3,11)	45,45 ^a^ (3,83)	36,51 (2,45)	33,28 ^a^ (3,93)	30,93 (3,15)	27,21 (3,58)	24,55 (4,86)	18,86 (3,71)	21,91 (6,53)	14,96 (5,55)
**nausea & vomiting**	6,34	8,84 (3,16)	13,31 (3,20)	4,70 (2,77)	7,20 (2,88)	1,47 (3,29)	4,67 (2,62)	3,56 (3,30)	−0,23 (3,55)	2,17 (5,99)	−2,00 (6,63)
**pain**	24,52	21,74 (3,68)	24,21 (3,45)	20,42 (3,39)	21,93 (3,93)	16,53 (4,35)	19,18 (3,55)	17,85 (5,34)	15,43 (4,11)	15,14 (7,29)	12,02 (7,38)
**dyspnea**	13,22	17,11 (3,28)	17,47 (2,45)	24,15 (2,89)	22,42 (4,08)	18,08 (3,71)	15,22 (3,62)	14,33 (4,65)	14,23 (3,97)	19,47 (4,53)	15,31 (5,67)
**insomnia**	30,85	29,74 (3,96)	30,25 (4,10)	33,59 (4,43)	31,43 (4,67)	28,77 (4,59)	26,19 (4,26)	29,74 (6,08)	19,74 (4,74)	32,94 (6,43)	26,69 (9,56)
**loss of appetite**	11,57	11,01 (4,00)	20,66 (4,36)	8,84 (3,84)	11,57 (3,18)	0,46 (4,48)	6,90 (2,86)	11,57 ^a^ (6,19)	1,47 (4,23)	11,57 (8,05)	−5,10 (5,27)
**obstipation**	11,85	14,07 (3,44)	19,12 (4,03)	12,94 (3,89)	18,74 (3,55)	14,62 (4,95)	9,85 (3,86)	5,18 (3,71)	9,83 (4,34)	7,68 (7,98)	−2,74 ^a^ (5,24)
**diarrhea**	5,23	8,01 (2,89)	8,26 (1,99)	9,06 (3,41)	9,83 (2,52)	6,62 (2,97)	5,90 (1,78)	9,68 (3,48)	1,19 (1,92)	5,23 (0,00)	7,32 (4,78)
**financial difficulty**	11,29	15,18 (2,25)	12,51 (2,59)	14,57 (2,88)	11,87 (2,24)	15,46 (2,74)	8,63 (2,99)	14,63 (6,26)	5,23 (4,22)	15,46 (4,17)	17,54 ^a^ (7,59)

^a^ Indicates more or equal to ten-point difference from previous time point.

T0: baseline, T1: last day RT, T2: 3 months post-RT, T3: 1 year post-RT, T4: 2 years post-RT, T5: 3 years post-RT.

**Table 5:** **EORTC QLQ**-**BR23 mean scores** (**SE**) **at each time point**

	**CR & TT at T0**	**CR at T1**	**TT at T1**	**CR at T2**	**TT at T2**	**CR at T3**	**TT at T3**	**CR at T4**	**TT at T4**	**CR at T5**	**TT at T5**
	**n=121**	**n=62**	**n=59**	**n=62**	**n=59**	**n=50**	**n=51**	**n=30**	**n=36**	**n=16**	**n=18**
**arm symptoms**	24,33	21,00 (2,50)	18,07 (2,32)	21,24 (2,82)	18,01 (2,08)	22,02 (3,73)	26,33 (4,27)	19,89 (5,10)	19,96 (4,40)	11,14 (5,76)	18,78 (6,49)
**breast symptoms**	20,94	33,30 ^a^ (2,78)	29,57 (2,74)	23,67 (2,58)	24,10 (2,35)	17,99 (3,17)	15,94 (2,52)	14,55 (4,24)	12,60 (3,30)	6,87 (5,63)	16,77 (4,99)
**body image**	73,35	69,60 (2,14)	70,62 (2,49)	73,21 (2,48)	77,80 (2,55)	74,74 (3,67)	77,51 (2,87)	73,62 (5,76)	78,40 (4,93)	74,91 (7,02)	79,08 (7,36)
**future perspective**	53,44	57,33 (3,64)	51,02 (3,44)	58,36 (3,38)	58,62 (3,26)	61,78 (4,03)	65,44 (3,53)	64,55 (5,84)	64,55 (6,27)	59,69 (7,59)	68,03 (10,08)
**systemic treatment side effects**	14,64	22,50 (1,87)	25,81^a^ (2,83)	22,52 (2,06)	21,21 (2,88)	18,01 (2,20)	17,88 (2,57)	19,40 (3,19)	13,20 (2,95)	17,62 (4,80)	15,24 (4,28)
	**n**=**21**	**n**=**22**	**n**=**26**	**n**=**16**	**n**=**20**	**n**=**8**	**n**=**10**	**n**=**8**	**n**=**6**	**n**=**3**	**n**=**3**
**upset by hair loss**	34,92	48,25 ^a^ (8,16)	27,51 (9,26)	23,81 ^a^ (11,11)	40,48 ^a^ (5,56)	46,02 ^a^ (11,10)	28,25 ^a^ (16,33)	1,62 ^a^ (0,00)	23,81 (29,40)	34,92 ^a^ (0,00)	34,92 ^a^ (0,00)
	**n**=**104**	**n**=**53**	**n**=**50**	**n**=**53**	**n**=**53**	**n**=**42**	**n**=**45**	**n**=**26**	**n**=**30**	**n**=**13**	**n**=**15**
**sexual functioning**	23,85	21,02 (2,68)	23,52 (3,45)	24,48 (3,04)	22,60 (3,36)	24,65 (3,50)	24,59 (2,90)	21,85 (4,44)	27,19 (3,61)	25,14 (6,92)	21,63 (5,36)
	**n**=**61**	**n**=**23**	**n**=**23**	**n**=**29**	**n**=**32**	**n**=**24**	**n**=**29**	**n**=**15**	**n**=**19**	**n**=**8**	**n**=**9**
**sexual enjoyment**	55,74	53,98 (5,39)	47,40 (3,31)	48,16 (5,77)	50,61 (5,45)	61,29 ^a^ (4,86)	49,94 (4,98)	64,07 (5,98)	48,59 (5,16)	62,40 ^a^ (6,67)	46,21 (9,52)

^a^ Indicates more or equal to ten-point difference from previous time point.

T0: baseline, T1: last day RT, T2: 3 months post-RT, T3: 1 year post-RT, T4: 2 years post-RT, T5: 3 years post-RT.

All functional scores and the global health status score in both treatment arms were temporarily decreased on the last day of radiotherapy (Figures 2a–f, Table 4), and subsequently improved over time, except for cognitive functioning in CR patients. On the last day of radiotherapy, the global health score was significantly worse in TT patients than CR patients (p = 0.0287) and the social functioning score was worse in TT patients than CR patients, but this difference was not significant (p = 0.0635). However, analysis using repeated measurements of ANOVA with the Bonferroni correction did not show any significant differences in these scores between treatment arms. At 3 months post-radiotherapy, there were clinically meaningful increases in the role- and social-functioning scores in TT patients (10.8 points for each score, Table 4). During the period from 3 months to 2 years post-radiotherapy, there were faster improvements in the physical-, cognitive-, and emotional-functioning scores in TT patients than CR patients, but these differences were not significant (Figures 2a, 2c, 2d). Figures 2a–f show that TT patients experienced greater long-term improvements than CR patients in global health status and in all functioning scores except for social functioning, but these differences were not significant.

Figures 3a–i show that both treatment arms had the same patterns of symptoms. Fatigue, nausea and vomiting, and constipation were increased on the last day of radiotherapy and subsequently decreased over time; pain had already decreased on the last day of radiotherapy and subsequently decreased further over time; and dyspnea, insomnia, diarrhea, and financial difficulty fluctuated during the follow-up period. There were clinically meaningful increases in fatigue scores in both treatment arms on the last day of radiotherapy (10.6 points in CR patients and 13.1 points in TT patients, Table 5). The fatigue scores in both treatment arms subsequently decreased, with a clinically meaningful reduction in TT patients at 3 months (12.2 points, Table 5). Figure 3a shows that the fatigue score eventually recovered better in TT patients than CR patients.

Figure 4a shows that the arm symptoms scores had already decreased in both treatment arms on the last day of radiotherapy. This score continued to decrease in CR patients, whereas it was higher in TT patients at 1 year post-radiotherapy, but this increase was not significant.

Both treatment arms had the same breast symptom and systemic side effect patterns during the follow-up period (Figures 4b and 4c). On the last day of radiotherapy, there were clinically meaningful increases in breast symptom scores in CR patients (12.4 points) and in systemic side effect scores in TT patients (11.2 points), and these scores subsequently decreased over time. At 3 years after the completion of radiotherapy, the breast symptom scores were increased in TT patients and continued to decrease in CR patients, but this difference between treatment arms was not clinically meaningful (9.9 points, Table 5). The systemic side effects scores were still higher than baseline in both treatment arms at 3 years after radiotherapy. The degree of hair loss is incorporated into the systemic side effects score. Not all patients reported hair loss. Figure 4d shows a fluctuating hair loss score in both treatment arms.

Figures 5a and 5b show that there were no clinically meaningful changes in body image or future perspective scores in either treatment arm. Both scores were slightly decreased on the last day of radiotherapy in both treatment arms, and subsequently improved over time.

Patients were given the option to decline answering the entire section on sexual functioning, or any part of it. Therefore, only patients who answered this section were included in the analysis. The question regarding sexual enjoyment was only asked if the patient indicated that they had been sexually active, and only a relatively small proportion of patients answered this question (Table 5).

Figure 5c shows relatively stable sexual functioning scores in both treatment arms, which is in accordance with the relatively stable body image and future perspective scores over time. As only a small number of patients answered the sexual enjoyment question, it is difficult to draw any conclusions about trends in this score (Figure 5d). Even though the sexual functioning scores were stable in both TT and CR patients, the sexual enjoyment score increased in CR patients and slowly decreased in TT patients.

## Discussion

This is the first study to compare HRQOL between two adjuvant radiotherapy approaches for breast cancer, CR and TT. In November 2011, the median post-radiotherapy follow-up time was 26 months (range 4–50 months).

Table 6 lists the recent studies comparing CR with TT. Most of these studies reported toxicity and control rates, and a few reported on cosmesis [15,19,20] and HRQOL [19]. In this study, we analyzed all five functioning scores and nine symptom sclores in the QLQ C-30 questionnaire and all four functioning scores and four symptom scores in the QLQ BR23 questionnaire. The UK Standardisation of Breast Radiotherapy (START) trials A and B [17-19] presented only three of the QLQ BR23 scores in their analysis: breast symptoms, arm symptoms, and body image. 

**Table 6:** Hypofractionated radiotherapy studies

**Trial**	**Period**	**n**	**Hypofraction schedule**	**SIB**	**Mastectomy**	**Regional nodes**	**IMRT/ IGRT**	**Chemotherapy**	**Cosmesis**	**HRQOL**
UK Start A [17,19]	1998-2002	2236	3 Gy x 13 F/ 5 weeks	No	Yes	Yes	NS	Yes	Yes	Yes
			3.2 Gy x 13 F/ 5 weeks							
UK Start B [18,19]	1999-2001	2215	2.67 Gy x 15 F/ 3 weeks	No	Yes	Yes	NS	Yes	Yes	Yes
Ontario [15]	1993-1996	1234	2.66 Gy x 16 F/ 3 weeks	No	No	No	NS	Yes	Yes	No
Egypt NCI [20]	2002-2003	30	2.66 Gy x 16 F/ 3 weeks	No	No	No	NS	No	Yes	No
UK FAST [21]	2004-2007	915	5.7 Gy x 5 F/ 5 weeks	?	No	No	Yes	No	No	No
			6 Gy x 5 F/ 5 weeks							
Hopital Necker (*) [22]	1982-1984	230	5.75 Gy x 4 F/ 17 days	No	Yes	?	NS	Yes	No	No
Royal Marsden Hospital [23]	1986-1998	1410	3 Gy x 13 F/ 5 weeks 3.3 Gy x 13 F/ 5 weeks	No	No	Yes	NS	No	No	No
Lahore [24]	1998-2004	300	5.4 Gy x 5 F/ 1 week 3.5 Gy x 10 F/ 2 weeks	No	Yes	Yes	NS	Yes	No	No
			2.66 Gy x 15 F/ 3 weeks							
UZ Brussel	2007-2011	121	2.8 Gy [SIB 3.4 Gy]x15 F/ 3 weeks	Yes	Yes	Yes	Yes	Yes	No	Yes

NS: not stated.

As expected in breast cancer patients receiving radiotherapy, patients in both treatment arms experienced a decrease in global health status score and all functioning scores on the last day of radiotherapy (Figures 2a–f, Table 4). This is consistent with the findings of the randomized study by Whelan et al. [39]. However, another small study conducted by Lee et al. [38] reported that radiotherapy did not affect the global health score compared with no radiotherapy in a randomized trial. In our study, the reasons for the decrease in global health score were most likely increased fatigue, breast symptoms, systemic side effects, nausea and vomiting, and loss of appetite, especially when patients received concomitant chemotherapy. This decrease in scores on the last day of radiotherapy was approximately the same in both treatment arms, except that TT patients had significantly worse global health status scores and non-significantly worse social functioning scores than CR patients. This difference might be due to more fatigue, nausea and vomiting, loss of appetite, and systemic side effects in TT patients than CR patients the end of radiotherapy (Figures 2 and 3, Table 4). This might be partially explained by the higher proportion of TT patients who received concomitant chemotherapy (39%) compared with CR patients (30%).

Fortunately, the decreases in global health status and functioning scores were only temporary, and these scores subsequently improved during the follow-up period, except that CR patients continued to have worse cognitive functioning at 3 years post-radiotherapy (Figures 2a–f).

At 3 months post-radiotherapy, there were clinically meaningful increases in role- and social-functioning scores in the TT group (Table 4). During the period from 3 months to 2 years post-radiotherapy, there were faster improvements in the physical-, cognitive-, and emotional- functioning scores in TT patients than CR patients (Figures 2a, 2c, 2d). No specific reason was identified for these (non-significant) differences, except that CR patients were slightly older than TT patients (mean age 58 years vs. 55 years). The proportion of patients aged > 65 years was 34% in the CR group and 22% in the TT group.

At 3 years post-radiotherapy, there were greater improvements in the global health status score and all functioning scores (except social functioning) in TT patients than CR patients, but these differences were not significant. Physical-, role-, and cognitive-functioning scores were between 5.0 and 9.4 points higher in TT patients than CR patients (Table 4).

After a temporarily increasing on the last day of radiotherapy, the fatigue scores in both treatment arms decreased during the follow-up period. This is consistent with the findings of other studies [38-42] in which fatigue was the most commonly reported symptom after radiotherapy. The increase in the fatigue score on the last day of radiotherapy was clinically meaningful in both treatment arms. This score had already decreased at 3 months post-radiotherapy in both treatment arms, and the decrease in TT patients was clinically meaningful (Table 5). There were no significant differences in fatigue scores between treatment arms at any time points. As mentioned above, fatigue was one of the factors causing decreased global health status and functioning scores. It has been reported that exercise is effective in helping to overcome fatigue during radiotherapy. Patients who exercise during radiotherapy have better physical functioning and less fatigue, anxiety, and insomnia than patients who do not exercise [43,44].

The HRQOL questionnaires were completed by CR patients an average of 42 days after surgery and by TT patients an average of 47 days after surgery. Patients were not yet fully recovered from their breast surgery at that time, which could explain the higher pain and arm symptom scores at baseline. Axillary node dissection was more frequent in TT patients (49%) than CR patients (31%), but the arm symptom scores were comparable between treatment arms (CR: 23.8 (± 22.6) vs. TT: 24.9 (± 21.6). The arm symptom scores had already decreased in both treatment arms on the last day of radiotherapy, and subsequently continued to decrease in CR patients, whereas the score was higher at 1 year post-radiotherapy in TT patients. None of these differences were significant. Our findings are in accordance with those of the START trial [19], which found that arm symptom scores were highest at baseline and then decreased significantly, and that there were no significant differences in scores between the treatment arms.

Both treatment arms had quite a large increase in breast symptom scores on the last day of radiotherapy, which was clinically meaningful in CR patients, and these scores subsequently decreased over time. These findings are consistent with the common acute side effects of radiotherapy, and are normally transient [38,39,45-47]. There was a greater increase in the breast symptom score on last day of radiotherapy in CR patients than TT patients, but this difference was not significantly different. This could partially be explained by the daily positioning at mm-level by the tomotherapy system [48]. Taher et al. [20] also found no significant differences in acute skin reactions or cosmetic appearance between the two treatment arms. The START trial [19] found that the BR23 breast symptom score declined significantly from baseline to 5 years for all radiotherapy regimens, but there was no significant difference between treatment arms. A randomized trial by Whelan et al. [15] which compared CR and TT schedules used the EORTC Cosmetic Rating System to measure late radiation toxicity. They concluded that the more convenient hypofractionated schedule appeared to be an acceptable alternative to CR. They found no differences between the treatment arms at 3 and 5 years after randomization, and a comparable cosmetic outcome at 10 years after treatment [16]. Ongoing follow-up in our study group will determine long-term breast symptom scores in both treatment arms, which will be reported in the future.

The systemic side effects scores were increased on the last day of radiotherapy in both treatment arms, and the increase was clinically meaningful in TT patients. This increase was most likely due to concomitant chemotherapy. There was a subsequent slow decrease in this score in both treatment arms. However, this score in was still higher than baseline at 3 years post-radiotherapy in both treatment arms. This could be explained by the administration of hormonal therapy to most patients for 5 years (86% of CR patients and 81% of TT patients) and the administration of herceptin to some patients for 1 year (5% of CR patients and 17% of TT patients) after the completion of radiotherapy.

Both treatment arms shared almost the same pattern of body image and future perspective scores, and there were no significant differences between groups at any of the time points. Even though patients had undergone MA or segmentectomy, and some patients had also undergone chemotherapy, these scores decreased only slightly at the end of radiotherapy in both treatment arms, and subsequently improved and then remained stable over time. This was consistent with the relatively stable sexual functioning scores in both treatment arms during the follow-up period. Our findings are consistent with those of the START trial [19], which found that body image scores were similar between treatment arms over time. They also found a significant improvement in body image score in all treatment arms over time, compared with the baseline score.

## Limitations

The HRQOL questionnaire provides patient-reported symptom and functional status, and enhances clinical decision making by considering the benefits and toxicity of treatment [49]. The EORTC QLQ-C30 and BR23 questionnaires were included in this randomized trial of CR and TT to provide further information. The primary endpoint of the trial was pulmonary or cardiac toxicity, and the secondary endpoint was locoregional recurrence. This trial has some limitations. First, the sample size is smaller at 2 and 3 years post-radiotherapy than at earlier time points, as the median follow-up time is 26 months (range 4–50 months). This limits the ability to draw conclusions regarding HRQOL at these time points. However, the available questionnaire results are presented in the tables and figures to illustrate trends, especially as one of the main concerns regarding radiotherapy is long-term toxicity. The final results of the trial can be reported after all patients have completed 3 years of follow-up after radiotherapy.

Second, some data are missing due to various reasons: withdrawal of patients from the study, refusal by several patients to complete the questionnaire on the last day of radiotherapy, and reluctance by patients to answer sex-related questions. In an ideal situation, there would be 100% compliance in questionnaire completion at all time points, and the repeated measurements of ANOVA could be used for analysis. Since ANOVA only takes patients with complete datasets into account, there would have to be no missing values or withdrawals from the study before 3 years of follow-up had been completed. In this study with incomplete data and a limited number of patients at 2 and 3 years of follow-up, the simpler Student’s t-test was used to compare HRQOL scores between the treatment arms. ANOVA did not show any significant differences between treatment arms.

Third, only the global health status score on the last day of radiotherapy was found to be significantly different between treatment arms (p = 0.0287). However, when the Bonferroni correction for multiple testing was applied, this difference was no longer significant. This could be explained by the small sample size, as a larger sample size may be needed to demonstrate significant differences.

Fourth, information regarding sociodemographic factors (marital status, income, occupation, etc.), which has been found to be related to QOL in cancer patients, was not gathered. Such sociodemographic factors should be considered in future trials, especially when evaluation of HRQOL is the main objective.

## Conclusion

Our study is the first to compare HRQOL between CR and TT using the Tomotherapy® treatment system. We found that TT patients had a faster improvement in QOL, role- and cognitive-functioning, and fatigue after radiotherapy than CR patients. The inconvenience of prolonged daily treatments substantially contributes to the decreased QOL in breast cancer patients treated with radiotherapy. Our results confirm that radiotherapy using a shorter fractionation schedule may reduce the burden of treatment and have important QOL benefits for breast cancer patients.

This research was funded by the Foundation against Cancer, a public interest foundation (SCIE2006-30, ref.nr ANI47).

## Competing interests

The authors declare that they have no competing interests.

## Authors' contributions

VVH and GS made substantial contributions to the conception and design of the study. VVH, HV, HVP, GM, MV, and NA made substantial contributions to the acquisition of data. HV and VVH made substantial contributions to the analysis and interpretation of data, and were involved in drafting the manuscript. VVH, HV, GS, and MDR critically revised the manuscript for important intellectual content. All authors read and approved the final manuscript.

## Pre-publication history

The pre-publication history for this paper can be accessed here:

http://www.biomedcentral.com/1471-2407/12/495/prepub

## Supplementary Material

Additional file 1 adjusted_mean_scores.xls. Click here for file
